# Anisakid Presence in the European Conger, *Conger conger*, from Spanish Mediterranean Waters †

**DOI:** 10.3390/pathogens12111326

**Published:** 2023-11-08

**Authors:** Xavier Roca-Geronès, Lídia Sarrà-Alarcón, Eulàlia Delgado, Maria Magdalena Alcover, Margarida Casadevall, Roser Fisa

**Affiliations:** 1 Secció de Parasitologia, Departament de Biologia, Sanitat i Medi Ambient, Facultat de Farmàcia i Ciències de l’Alimentació, Universitat de Barcelona, 08028 Barcelona, Spain; 2 Department de Ciències Ambientals, Facultat de Ciències, Universitat de Girona, 17003 Girona, Spain

**Keywords:** Conger, *Anisakis*, nematode, prey, Mediterranean

## Abstract

The European conger, *Conger conger*, is a benthic marine fish species with a geographical distribution extending through the northeastern Atlantic and the Mediterranean. Despite being extensively distributed and widely appreciated by Spanish consumers, studies regarding parasite presence in this fish are scarce. In the present work, a hundred and eight specimens from the Mediterranean coast of northeastern Spain (Catalan waters) were surveyed for the presence of nematode parasites. Several species were morphologically identified: third-stage larvae of *Anisakis* type I (sensu Berland, 1961) (*n* = 131), third-stage larvae of *Anisakis* type II (sensu Berland, 1961) (*n* = 18), third- and fourth-stage larvae and adults of *Hysterothylacium* spp. (*n* = 48), adults of *Cucullanus* sp. (*n* = 391), and adults of *Cristitectus congeri* (*n* = 69). Moreover, some fish and decapode species were also observed as part of the host’s diet, with the most detected preys being *Micromesistius poutassou*, *Sardina pilchardus*, *Macropipus* sp., and *Goneplax rhomboides*. This represents the first survey of nematode parasites infecting *C. conger* from the northeastern Spanish Mediterranean waters. Among the parasite species detected, the presence of *Anisakis* species should be highlighted as the ingestion of *C. conger* parasitized with these larvae could potentially lead to anisakiasis in consumers.

## 1. Introduction

The European conger, *Conger conger* (Linnaeus, 1758), is a benthic marine fish species inhabiting rocky and sandy bottoms between 5 to 1000 m in depth and presenting sex segregation in some areas [1,2,3]. It is a large opportunistic predator, with a diet including a wide range of crustaceans, fish, and cephalopods [4], which vary in relation to the host’s living depth and its reproductive cycle [5].

The geographical distribution of the European conger extends through the northeastern Atlantic, from Norway and Iceland to Senegal, including the Macaronesian archipelagos, and the Mediterranean and Black Seas [6]. This species is of great importance in commercial and recreational fisheries on both Atlantic and Mediterranean coasts [6,7]. In 2021, the total world catches of *C. conger* were 9480.6 t [8], with Spain being one of the countries with the highest number of catches through the years [9,10].

Studies regarding the parasite fauna of *C. conger* in the Mediterranean Sea and the northeastern Atlantic have identified several species corresponding to different phyla. Some of the nematodes identified include *Anisakis* Dujardin, 1845*, Cucullanus* Müller, 1777 or *Cristitectus* Petter, 1970 species. Regarding digenids, *Helicometra* Odhner, 1902, *Lecithochirium* Lühe, 1901 and *Prosorhynchus* Odhner, 1905 species, among others, have been detected, while Tetraphillidids and Trypanorhyincha species have been observed corresponding to cestodes [11,12,13,14,15].

Among all these species, the European Food Safety Authority (EFSA) considers *A. simplex* s.l. as an important “biological hazard” in seafood products [16]. This species can cause anisakiasis in humans when raw or undercooked fish is consumed, provoking severe gastrointestinal and/or allergic symptoms [17,18]. In Spanish waters, the parasite community of the European conger has been poorly studied, despite being extensively distributed and widely appreciated by consumers.

The aim of the present study was to survey the commonly consumed *C. conger* from the Mediterranean coast of northeastern Spain (Catalan waters) for the presence of nematode species. Moreover, special emphasis was placed on analyzing the content of the host’s stomach to analyze its diet in this area.

## 2. Materials and Methods

### 2.1. Host Sampling and Parasite Collection

A parasitological analysis focused on nematode species was carried out on 108 specimens of *C. conger* collected during the years 2011–2012 from the Catalan Mediterranean coasts, in the northeastern Spanish waters, corresponding to the FAO (Food and Agriculture Organization of the United Nations) fishing area 37.1.1. After measuring total length and weight, fish were dissected and weighed again. The total length, total weight, and eviscerated weight of the 108 European conger (*C. conger*) specimens analyzed are shown in Table 1. The viscera were isolated and organs including the stomach, intestine, liver, and gonads were separated in petri dishes containing physiological saline solution. Each part was examined for the presence of nematodes under a stereomicroscope using a fiber optical illuminator. In addition, the external part of the musculature surrounding the host’s belly flaps was also inspected.

Parasites were morphologically identified by observing the main structures of their anterior and posterior ends. The presence of a tooth, the shape of the ventricle, and the morphology of the posterior end were used to identify *Anisakis* and *Hysterothylacium* Ward & Magath, 1917 species following the criteria of Berland [19] and Hartwich [20]. For the identification of *Cucullanus*, the shape of the anterior end, the form of the oesophagus, and the sexual structures of the caudal end were used according to Berland [19]. Finally, following Petter’s [21] and Quinteiro et al.’s [22] criteria, species of *Cristitectus* were identified by examining the morphology of their anterior end, containing the cuticular ridges, and the posterior end.

### 2.2. Host Diet

The host’s stomach samples, including the entire contents, were weighed after the removal of surface water by blotting with tissue paper. Prey items were carefully separated, counted, and weighed individually. All components were divided into fish (Osteichthyes) or crustacean (Decapoda) and identified to the lowest taxonomic level possible using the identification guides and keys provided by Zariquiey [23] and Fischer et al. [24]. When it was impossible to identify the prey at the species level due to missing parts, classification was carried out at the next level (for example, family). Fragmented prey was counted using a part of their body that was easy to determine (for example, telson for Decapodes). If the prey was too fragmented, it was considered as a single prey. A group of not-determined prey was included for each taxonomic group when the prey presented advanced levels of digestion.

### 2.3. Epidemiological Concepts and Statistical Analysis

Estimated prevalence (P), mean intensity, and mean abundance (A), as descriptive epidemiological terms [25], were calculated using the Quantitative Parasitology Software 3.0 [26] in the 95% confidence interval (CI) for each parameter (2000 bootstrap replications).

Spearman’s Rho (Rho) nonparametric test was applied to analyze the correlation between larval abundance and fish total length and weight. All parameters and variables were tabulated for their analysis in SPSS v22 software.

## 3. Results

### 3.1. Host Parasitic Infection

**Figure 1:**
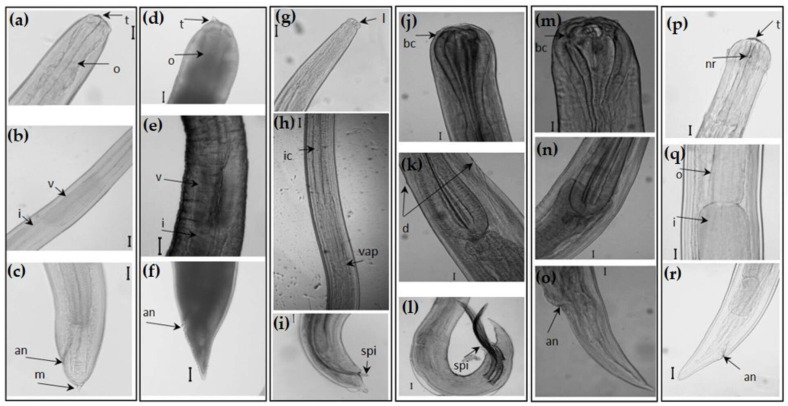
Microphotographs showing anterior, central, and posterior regions of the nematodes found in *C. conger* specimens: (**a**–**c**) anterior (**a**), central (**b**), and posterior (**c**) regions of L3 of *Anisakis* type I (sensu Berland, 1961); (**d**–**f**): anterior (**d**), central (**e**), and posterior (**f**) regions of L3 of *Anisakis* type II (sensu Berland, 1961) (**g**–**i**): anterior (**g**), central (**h**), and posterior (**i**) regions of an adult of *Hysterothylacium* spp.; (**j**–**l**): anterior (**j**), central (**k**), and posterior (**l**) regions of an adult male of *Cucullanus* sp.; (**m**–**o**): anterior (**m**), central (**n**), and posterior (**o**) regions of an adult female of *Cucullanus* sp.; (**p**–**r**): anterior (**p**), central (**q**), and posterior (**r**) regions of an adult of *C. congeri*. t: tooth, o: esophagus, v: ventricle, i: intestine, m: mucron, an: anus, l: labia, ic: intestinal caecum, vap: ventricular appendix, spi: spicules, bc: buccal capsule, d:deirids, nr: nerve ring; Scale bar 50 µm in all microphotographs with the exception of b, h and l (100 µm).

The nematode analysis of the host’s viscera resulted in the identification of 657 specimens, which were morphologically identified as third-stage larvae (L3) of *Anisakis* type I (sensu Berland, 1961) (*n* = 131), L3 of *Anisakis* type II (sensu Berland, 1961) (*n* = 18), adults of *Cucullanus* sp. (*n* = 391), adults of *Cristitectus congeri* Petter, 1970 (*n* = 69), and species of *Hysterothylacium* (*n* = 48) (Figure 1). Specimens of *Hysterothylacium* were found in three different evolutionary stages, L3 (*n* = 7), fourth-stage larvae (L4) (*n* = 38), and adults (*n* = 3). Nematodes were mostly found in the visceral cavity, including the stomach, the intestine, and the liver, for species of *Anisakis* and *Cucullanus*, and the stomach and the intestine for specimens of *Hysterothylacium* spp. and *C. congeri*. Moreover, some specimens were also found encysted on the surface of the musculature surrounding the viscera.

The analysis results of the descriptive epidemiological variables of host parasitisation by nematodes are shown in Table 2. *Cucullanus* sp. and *Anisakis* type I (sensu Berland, 1961) species were the most prevalent parasites (P = 79.6% and P = 33.3%, respectively) with mean abundance values of 3.6 for the former species and 1.2 for the latter.

Spearman’s correlation analysis showed a significant positive correlation (*p* < 0.01) between host length and weight and the total abundance of all nematodes identified (ρ = 0.4 for both parameters). When comparing the two biometric parameters with the number of specimens of the different nematode species, a significant positive correlation (*p* < 0.01) was detected for *Anisakis* type I (sensu Berland, 1961) (ρ = 0.5), *Anisakis* type II (sensu Berland, 1961) (ρ = 0.3), and *Cucullanus* sp. (ρ = 0.3).

### 3.2. Host Prey Items

A total of 78 individuals of European conger contained food in their stomachs (72.2%). Fish items (*n* = 79) were found in 53 *C. conger* specimens (49.1%) while crustacean remains (*n* = 65) were observed in 35 hosts (32.4%). Among them, a total of 40 prey taxa were identified, with 24 belonging to osteichthyes and 16 to decapods (Table 3). Regarding fish species, *Micromesistius poutassou* (Risso, 1827) (*n* = 15) and *Sardina pilchardus* (Walbaum, 1792) (*n* = 8) were the most frequent prey while 32 remains (40.52%) could not be determined. *Macropipus* Prestandrea, 1833 specimens (*n* = 22) and *Goneplax rhomboides* (Linnaeus, 1758) (*n* = 8) were the most detected crustaceans, with 19 decapod remains (29.2%) unable to be identified.

## 4. Discussion

Several nematode species have been identified parasitizing the European conger in Mediterranean and Atlantic waters [12,14]. In the present study, we report for the first time the identification of five nematode species in *C. conger* specimens from the Mediterranean coast of northeastern Spain (Catalan coast), corresponding to *Anisakis* type I (sensu Berland, 1961), *Anisakis* type II (sensu Berland, 1961), *Hysterothylacium* spp., *Cucullanus* sp., and *C. congeri* (Table 2). The four former species have been identified in a wide range of hosts worldwide, including several fish and cephalopod species [27,28,29,30,31], while *C. congeri* is known to be a specialist nematode of conger eels [21,22]. The *Anisakis* larvae found in the studied fish were denominated *Anisakis* type I and type II (sensu Berland, 1961) as they were only identified following morphological criteria. However, specimens of *Anisakis* type I (sensu Berland, 1961) probably correspond to *Anisakis pegreffii* Campana-Rouget & Biocca, 1955, included in the *A. simplex* sensu lato complex, as it is the most frequent species of the complex identified on the Catalan coast [32,33,34]. In the same line, molecular methodologies could confirm the identification of *Anisakis* type II as *Anisakis physeteris* Baylis, 1923 [32,33,34].

In other regions of the Spanish Mediterranean area, only Muñoz et al. [35] from the central Eastern coast (Valencian waters) have analyzed the parasitological fauna of *C. conger*, identifying *Cucullanus hians* (Dujardin, 1845) Petter, 1974, *Cucullanus longispiculum* De Oliveira Rodrigues, Carvalho Varela, Sodre’ Rodrigues & Cristofaro, 1973 and species of *Dichelyne* Jägerskiöld, 1902. For both species of the genus *Cucullanus*, prevalence (58% and 43%, respectively) and mean intensity (2 for both species) values were lower in comparison with the results obtained in the present work (79.6% and 4.6 for *Cucullanus* sp.). It should be noted that, despite the geographical area, where anisakid nematodes are habitually identified in other fish species such as the blue whiting or the surmullet [36,37,38], and the high number of hosts analyzed (78), species of *Anisakis* or *Hysterotylacium* were not detected [35].

In other Mediterranean areas, such as the Sardinian waters and the Aegean Sea, nematode species detected in our work were also identified [14,36]. In Sardinia, values of prevalence and mean abundance for *Anisakis simplex* s.l. (P = 11.5; A = 0.1), *Cucullanus bioccai* Orecchia & Paggi, 1987 (P = 46.2; A = 2.9), and *C. congeri* (P = 7.7; A = 0.1) were lower compared to those observed in the present study (Table 2). On the other hand, *Hysterothylacium* spp. (P = 61.5; A = 2.0) presented higher values in comparison with those herein obtained (P = 26.9; A = 0.4). The presence of *A. physeteris* was scarce in both Mediterranean areas [14]. In the Aegean Sea, the nematode species identified corresponded only to *A. simplex* s.l. and *C. hians* [39]. For both species, prevalence was lower (15.38% and 7.69%, respectively) while mean intensity was higher (3.5 and 6, respectively) compared to the values of our work (Table 2). It is noteworthy that the number of analyzed hosts in the current investigation was higher than the previous Mediterranean studies [14,35,39], standing out as representative in terms of nematode analysis of the European conger.

In the Atlantic area, including the Spanish coast and the Madeira Islands, several authors have identified *A. simplex* s.l. parasitizing *C. conger*, showing in all cases lower prevalence (from 1.1% to 9%) and mean abundance values (from 0.01 to 1.3) [12,15,40]. These epidemiological values contrast with the remarkably high prevalence and mean abundance values of *A. simplex* s.l. observed in other fish species from the northeastern Atlantic waters [32,38,41]. On the other hand, in the Atlantic waters, prevalence and intensity of *Cucullanus* species and *C. congeri* were variable, detecting higher and lower values in comparison with our work [11,12,15,22,40].

The variability of the nematode epidemiological terms between areas could depend on the biotic and abiotic conditions of the host habitat, which could lead to particular environmental conditions [12,40]. Moreover, host population isolation and differences in their trophic chain could also be an important differentiation factor [40]. In this sense, the opportunistic predatory behavior of the *C. conger* was confirmed by the high variability detected between the prey items of the host diet, including a wide number of taxa for both fish and crustaceans (Table 3). This variability has also been observed in other Atlantic and Mediterranean areas, where fish and crustacean species identified in the host’s stomach were also very diverse [4,42,43,44], being the most detected prey items of our study (*M. poutassou*, *S. pilchardus*, *Macropipus* sp., and *G. rhomboides*) absent or very scarce.

The remarkable presence of *Anisakis* type I (sensu Berland, 1961) in the European conger of the present work (P = 33.3%) could be related to the ingestion of *M. poutassou,* which is known to be a fish species habitually infested with this parasite in the studied area [32,45,46]. On the other hand, *S. pilchardus*, the other fish species identified as a habitual prey of the European conger from the Catalan coast, is not frequently parasitized with *Anisakis* type I (sensu Berland, 1961) in this region [47,48].

From all of the nematode species identified in the present study, *Anisakis* type I (sensu Berland, 1961) is the most important in terms of public health as it can cause gastric and/or allergic anisakiasis in humans [17,18]. In Spain, several cases have been reported in recent years [49,50]. Most of the gastric and intestinal cases are associated with the consumption of marinated anchovies, a typical dish in Spanish cuisine. Additionally, other species such as hake and sardine have also been reported as a source of infection [50,51,52].

*Conger conger* has not been documented as the source of anisakiasis in northeastern Atlantic and Mediterranean countries, but it should be taken into account as a possible cause of the disease due to the notable detection of *Anisakis* type I (sensu Berland, 1961). The analysis carried out in the present work was focused on the viscera and the external part of the anteroventral muscle, which has been documented as the most parasitized portion of the flesh in several fish species, such as the blue whiting, the grey gurnard, or the beaked redfish [32,53,54]. Although the number of larvae decreases in the posterior part of the musculature [32,53,54], a deeper examination of the flesh using specific methodologies, e.g., the UV-press method or the pepsin digestion, might increase the number of *A. simplex* s.l. identified in the European conger herein studied.

Moreover, in South Korea, where the common conger species is *Conger myriaster* (Brevoort, 1856), several gastric and allergic anisakiasis cases have been documented after the raw ingestion of this fish [55,56]. In China, *A. simplex* s.l. has been detected in the same host species with high prevalence and intensity values [57].

The epidemiological results of the European conger herein obtained reinforce the consideration of *C. conger* in terms of health risk, as values are in some cases higher than some fish species from the Catalan coast, such as the blue whiting or the Atlantic mackerel, among others [32,48]. As occurring in other fish species [32,58], special emphasis should be placed on the biggest *C. conger* specimens, as the host’s length and weight have been positively correlated with *Anisakis* type I (sensu Berland, 1961) abundance. Furthermore, globalization has led to worldwide changes in eating habits, and together with the increasing consumption of fish products, especially raw forms like sushi or ceviche, the number of anisakiasis cases has also increased worldwide [59,60]. In this context, due to its extensive distribution and popularity, Spanish consumers should be warned of the risk of consuming raw or undercooked *C. conger*.

## 5. Conclusions

Our research provides the first parasite identification focused on nematode species in *C. conger* from the Mediterranean coast of northeastern Spain. *Anisakis* type I (sensu Berland, 1961) and *Cucullanus* sp. were the most frequent nematode parasites, presenting remarkable prevalence and mean abundance values. In this sense, Spanish consumers should be aware of the possible presence of *Anisakis* type I (sensu Berland, 1961) in Mediterranean European conger due to the gastrointestinal and/or allergic illness that this parasite can cause. The presence of this nematode species in the hosts herein studied might be related to the ingestion of *M. poutassou*, which was identified as one of the most habitual prey of the European conger. In addition, a wide number of fish and crustacean species were identified as prey items of the *C. conger* diet, confirming the opportunistic predatory behavior of this fish species.

## Figures and Tables

**Table 1:** Biometric parameters of the 108 *C. conger* analyzed.

Parameter	Mean ± SD	Range
Total length (cm)	75.3 ± 26.7	180.0–29.8
Total weight ^1^ (g)	1252.1 ± 1972.1	15,000.0–31.9
Eviscerated weight ^2^ (g)	976.3 ± 1293.7	8200.0–27.4

^1^ *n* = 107; ^2^ *n* = 106.

**Table 2:** Descriptive epidemiological results of *C. conger* infection with larvae of nematode species.

Parasite Specie	N	Prevalence % (95% CI)	Mean Intensity (95% CI)	Mean Abundance (95% CI)
*Anisakis* type I (sensu Berland, 1961)	131	33.3 (25.1–42.7)	3.6 (2.7–4.6)	1.2 (0.7–1.8)
*Anisakis* type II (sensu Berland, 1961)	18	10.2 (5.6–17.5)	1.6 (1.2–2.1)	0.2 (0.05–0.29)
*Cucullanus* sp.	391	79.6 (71.0–86.2)	4.5 (3.6–5.5)	3.6 (2.8–4.5)
*Cristitectus congeri*	69	13.0 (7.8–20.7)	4.9 (3.2–6.7)	0.6 (0.06–1.2)
*Hysterothylacium* spp.	48	26.9 (19.4–36.0)	1.7 (1.3–2.0)	0.4 (0.3–0.6)

**Table 3:** Fish and crustacean species detected in the stomach of the 78 *C. conger* specimens with food remains.

Species	N. of Preys (%)	Range
Fish-Osteichthyes		
*Dalophis imberbis* (Delaroche, 1809)	1 (1.3)	-
Species of *Serranus* Cuvier, 1816	1 (1.3)	-
*Serranus cabrilla* (Linnaeus, 1758)	2 (2.5)	1–1
*Merluccius merluccius* (Linnaeus, 1758)	1 (1.3)	-
*Mullus surmuletus* Linnaeus, 1758	1 (1.3)	-
*Mullus barbatus* Linnaeus, 1758	1 (1.3)	-
*Gnathophis mystax* (Delaroche, 1809)	1 (1.3)	-
Family Scombridae Rafinesque, 1815	1 (1.3)	-
*Micromesistius poutassou* (Risso, 1827)	15 (19.0)	1–4
*Sardina pilchardus* (Walbaum, 1792)	8 (10.1)	1–3
*Phycis blennoides* (Brünnich, 1768)	1 (1.3)	-
*Conger conger* (Linnaeus, 1758)	2 (2.5)	1–1
Family Argentinidae Bonaparte, 1846	1 (1.3)	-
*Argentina sphyraena* Linnaeus, 1758	2 (2.5)	- ^1^
Family Gonostomatidae Cocco, 1838	1 (1.3)	-
*Boops boops* (Linnaeus, 1758)	1 (1.3)	-
*Lepidorhombus boscii* (Risso, 1810)	1 (1.3)	-
*Peristedion cataphractum* (Linnaeus, 1758)	1 (1.3)	-
*Scyliorhinus canicula* (Linnaeus, 1758)	1 (1.3)	-
*Trachyrhynchus trachyrhynchus* (Risso, 1810)	1 (1.3)	-
Species of *Apterichtus* Duméril, 1806	1 (1.3)	-
*Pagrus pagrus* (Linnaeus, 1758)	1 (1.3)	-
*Diplodus sargus sargus* (Linnaeus, 1758)	1 (1.3)	-
Crustacea-Decapod		
*Scyllarus pygmaeus* (Spence Bate, 1888)	1 (1.5)	-
Species of *Processa* Leach, 1815	2 (3.1)	1–1
*Processa canaliculata* Leach, 1815	1 (1.5)	-
*Processa edulis* (Risso, 1816)	1 (1.5)	-
Species of *Macropipus* Prestandrea, 1833	11 (16.9)	1–3
*Macropipus puber* (Linnaeus, 1767)	11 (16.9)	1–4
*Liocarcinus depurator* (Linnaeus, 1758)	2 (3.1)	1–1
*Goneplax rhomboides* (Linnaeus, 1758)	8 (12.3)	1–4
*Solenocera membranacea* (Risso, 1816)	1 (1.5)	-
Species of *Alpheus* Fabricius, 1798	1 (1.5)	-
*Alpheus glaber* (Olivi, 1792)	1 (1.5)	-
*Alpheus macrocheles* (Hailstone, 1835)	1 (1.5)	-
*Galathea strigosa* Linnaeus, 1761)	1 (1.5)	-
*Munida intermedia* A. Milne-Edwards & Bouvier, 1899	2 (3.1)	- ^1^
*Aristeus antennatus* (Risso, 1816)	1 (1.5)	-
*Calappa granulata* (Linnaeus, 1758)	1	-

N.: number; ^1^ The two specimens were found in the same host.

## Data Availability

All relevant data are provided in the present study.
